# Clinical disease activity and acute phase reactant levels are discordant among patients with active rheumatoid arthritis: acute phase reactant levels contribute separately to predicting outcome at one year

**DOI:** 10.1186/ar4469

**Published:** 2014-02-03

**Authors:** Jonathan Kay, Olga Morgacheva, Susan P Messing, Joel M Kremer, Jeffrey D Greenberg, George W Reed, Ellen M Gravallese, Daniel E Furst

**Affiliations:** 1 Division of Rheumatology, Department of Medicine, University of Massachusetts Medical School and UMass Memorial Medical Center, 119 Belmont Street, Worcester, MA 01605, USA; 2 Division of Rheumatology, Department of Medicine, David Geffen School of Medicine, 10833 Le Conte Avenue, Los Angeles, CA 90095, USA; 3 Department of Biostatistics and Computational Biology, University of Rochester Medical Center, 601 Elmwood Avenue, Rochester, NY 14642, USA; 4 Division of Rheumatology, Department of Medicine, Albany Medical College and the Center for Rheumatology, 1367 Washington Avenue, Albany, NY 12203, USA; 5 Department of Rheumatology, New York University Hospital for Joint Diseases, 301 East 17th Street, New York, NY 10003, USA; 6 The Consortium of Rheumatology Researchers of North America, Inc. (CORRONA), 10 Vickery Hill Lane, Southborough, MA 01772, USA

## Abstract

**Introduction:**

Clinical trials of new treatments for rheumatoid arthritis (RA) typically require subjects to have an elevated acute phase reactant (APR), in addition to tender and swollen joints. However, despite the elevation of individual components of the Clinical Disease Activity Index (CDAI) (tender and swollen joint counts and patient and physician global assessment), some patients with active RA may have normal erythrocyte sedimentation rate (ESR) and/or C-reactive protein (CRP) levels and thus fail to meet entry criteria for clinical trials. We assessed the relationship between CDAI and APRs in the Consortium of Rheumatology Researchers of North America (CORRONA) registry by comparing baseline characteristics and one-year clinical outcomes of patients with active RA, grouped by baseline APR levels.

**Methods:**

This was an observational study of 9,135 RA patients who had both ESR and CRP drawn and a visit at which CDAI was >2.8 (not in remission).

**Results:**

Of 9,135 patients with active RA, 58% had neither elevated ESR nor CRP; only 16% had both elevated ESR and CRP and 26% had either ESR or CRP elevated. Among the 4,228 patients who had a one-year follow-up visit, both baseline and one-year follow-up modified Health Assessment Questionnaire (mHAQ) and CDAI scores were lowest for patients with active RA but with neither APR elevated; both mHAQ and CDAI scores increased sequentially with the increase in number of elevated APR levels at baseline. Each individual component of the CDAI followed the same trend, both at baseline and at one-year follow-up. The magnitude of improvement in both CDAI and mHAQ scores at one year was associated positively with the number of APRs elevated at baseline.

**Conclusions:**

In a large United States registry of RA patients, APR levels often do not correlate with disease activity as measured by joint counts and global assessments. These data strongly suggest that it is appropriate to obtain both ESR and CRP from RA patients at the initial visit. Requiring an elevation in APR levels as a criterion for inclusion of RA patients in studies of experimental agents may exclude some patients with active disease.

## Introduction

Among patients with rheumatoid arthritis (RA), disease progression over time is difficult to predict. Demographic, clinical, laboratory, and imaging parameters, both alone and in combination, have been used to predict outcomes. In some studies, erythrocyte sedimentation rate (ESR) elevation correlated with disease outcomes such as joint erosion [1-4] and Health Assessment Questionnaire Disability Index (HAQ-DI) scores [5] whereas, in others, increased C-reactive protein (CRP) levels (>20 mg/L) correlated better with radiographic [6-10] and functional [9,11] outcomes. However, when acute phase reactants (APRs) are discordant, with elevation of only one (ESR or CRP), these laboratory test results may not predict progression of structural damage to joints [12]. Combining clinical measures with an APR level into composite disease activity indices, such as the Disease Activity Score (DAS)28 or the Simplified Disease Activity Index (SDAI), often predicts structural and functional consequences [3,10,13-15]. However, joint destruction may continue to progress and functional status may still deteriorate in RA patients treated with nonbiologic disease-modifying antirheumatic drugs (DMARDs), despite both improvement in clinical parameters and persistent reduction of the ESR [12,16].

Clinical trials of new treatments for RA typically require subjects to have an elevated acute phase reactant, in addition to tender and swollen joints. However, despite the elevation of individual components of the Clinical Disease Activity Index (CDAI) (tender and swollen joint counts and patient and physician global assessment), some patients with active RA may have normal ESR and/or CRP levels [17-20] and thus fail to meet entry criteria for clinical trials [21].

In this study, we assessed the relationship between CDAI and APRs in the Consortium of Rheumatology Researchers of North America (CORRONA) database, a large United States registry of RA patients. We compared the baseline characteristics and one-year clinical outcomes of patients with active RA, grouped by baseline APR levels.

## Subjects and methods

### Definitions

The Clinical Disease Activity Index (CDAI) is a simplified disease activity score using a 28-joint count that integrates measures of physical examination, patient self-assessment, and evaluator assessment. The CDAI score (a scale of 0 to 76.0) is the numerical sum of the 28-joint tender and swollen joint counts and the patient and evaluator global health status, each assessed on a 10-cm visual analog scale [22]. It provides a validated indication of RA disease activity, with scores of ≤2.8 signifying remission and scores of >22 connoting high disease activity [23]. Because it requires no laboratory test result, the CDAI is easy to calculate and allows for immediate scoring.

The modified Health Assessment Questionnaire (mHAQ) is a simplified version of the Health Assessment Questionnaire (HAQ). The HAQ consists of 20 questions that inquire as to a patient’s ability to perform 20 activities of daily living. These questions are grouped into eight categories. The mHAQ consists of eight questions, with one taken from each category of the HAQ [24]. The total mHAQ score is calculated as the mean of the individual scores for each activity [25].

APR levels were considered to be elevated if the ESR was >28 mm/h or if the CRP was >8 mg/L (>0.8 mg/dL).

### Subjects

The CORRONA database from October 1, 2001, through February 27, 2011, was used to identify subjects for this study [26]. Ethical approval for participation in the CORRONA registry was obtained from the respective institutional review boards of participating academic recruitment sites (Bassett Healthcare Network Institutional Review Board, Cooperstown, NY; Baylor Research Institute Institutional Review Board, Dallas, TX; Colorado Multiple Institutional Review Board, Aurora, CO; Geisinger Health Systems Institutional Review Board, Danville, PA; Hospital for Special Surgery Institutional Review Board, New York, NY; Institutional Review Board of the University of Michigan Medical School, Ann Arbor, MI; Johns Hopkins Medicine Institutional Review Board, Baltimore, MD; KUMC Human Subjects Committee, Kansas City, KS; Medical College of Wisconsin/Froedtert Hospital Institutional Review Board, Milwaukee, WI; Meridian Health Institutional Review Board, Neptune, NJ; Montefiore Medical Center Institutional Review Board, Bronx, NY; North Shore-Long Island Jewish Institutional Review Board, Great Neck, NY; Northwestern University Institutional Review Board, Chicago, IL; NYU School of Medicine Institutional Review Board, New York, NY; Ochsner Institutional Review Board, New Orleans, LA; Rhode Island Hospital Institutional Review Board, Providence, RI; Saint Louis University Institutional Review Board, St. Louis, MO; Southern New Hampshire Medical Center Institutional Review Board, Nashua, NH; SUNY Downstate Institutional Review Board, Brooklyn, NY; UAB Institutional Review Board for Human Use, Birmingham, AL; University of California, Los Angeles Institutional Review Board, Los Angeles, CA; University of California, San Diego Institutional Review Board, San Diego, CA; University of Maryland, Baltimore Institutional Review Board, Baltimore, MD; University of Massachusetts Medical School Committee for the Protection of Human Subjects in Research, Worcester, MA; University of Rochester Research Subjects Review Board, Rochester, NY; University of Wisconsin-Madison Health Sciences Institutional Review Board, Madison, WI; UT Southwestern Institutional Review Board, Dallas, TX; Washington University in St. Louis Institutional Review Board, St. Louis, MO) and from the New England Institutional Review Board (Newton, MA), a central institutional review board, for community-based private recruitment sites. All patients provided written informed consent prior to enrollment in the cohort, which allowed all subsequent analyses of the anonymized data.

Patients were eligible for inclusion if they had a rheumatologist-confirmed diagnosis of RA, but not of psoriatic arthritis (PsA), and a visit with CDAI >2.8 (not in remission) and both an ESR and CRP drawn at the visit. A patient’s first visit in the CORRONA database with CDAI >2.8 and reported APR information was selected as the baseline visit. The CORRONA registry does not mandate measurement of either APR, but records the results of all laboratory studies ordered in the course of routine clinical care. The baseline visit could be either the enrollment visit into the CORRONA database or a follow-up visit. Drug initiation was not a criterion. A one-year follow-up visit was defined as a visit occurring 365 ± 60 days after the study baseline visit with both the mHAQ and CDAI measured.

A sensitivity analysis was performed in the subset of biologic-naïve RA patients, defined as those patients who had not yet used any biologic agent before or at the time of the baseline visit.

### Statistical methods

Comparisons of outcomes among APR groups (neither (zero) APR elevated, discordant APR levels (one APR elevated), or both (two) APR elevated) were assessed using linear regression models for most continuous outcomes. Log-linear models were used as noted in the tables for outcomes with distributional properties less appropriate for linear regression. Logistic regression models were used for comparison of binary outcomes. In all cases *P* values were generated using robust variance estimators (sandwich estimators), which provide a method that is not unduly affected by outliers or other small departures from model assumptions. Comparisons of CDAI category distributions were carried out using *χ*^2^ tests. No adjustments were made for multiple comparisons. All statistical analyses were carried out using SAS 9.3 on a Windows 7 platform (SAS Institute Inc., Cary, NC, USA).

## Results

### Baseline characteristics

**Figure 1:**
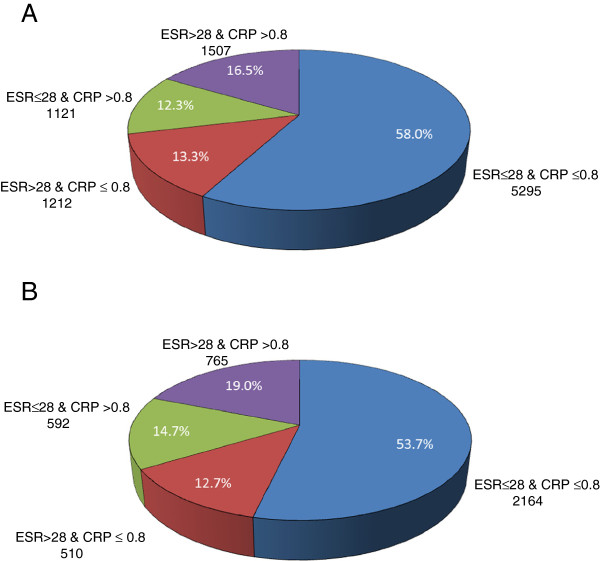
**Distribution of acute phase reactant levels at baseline.** Distribution of ESR and CRP levels at baseline **(A)** among the 9,135 patients who had a visit with active RA (CDAI >2.8), and **(B)** among the 4,031 biologic-naïve patients who had a visit with active RA (CDAI >2.8). CDAI, Clinical Disease Activity Index; CRP, C-reactive protein; ESR, erythrocyte sedimentation rate; RA, rheumatoid arthritis.

As of February 27, 2011, 27,412 patients with RA were enrolled in the CORRONA registry. Of these, 9,135 patients had a visit with active RA (CDAI >2.8) and had values recorded for both ESR and CRP and thus were eligible for sample inclusion. Of the patients with active RA, 5,295 (58.0%) had neither elevated ESR nor CRP. Only 1,507 (16.5%) had elevated levels of both ESR and CRP. Among the remaining 25.5% of patients, levels of ESR and CRP were discordant: 1,212 (13.3%) had an elevated ESR, but not CRP, and 1,121 (12.3%) had an elevated CRP, but not ESR (Figure 1A).

Among the 9,135 patients, 4,031 (44.1%) were biologic-naïve. The distribution of APR levels in this subset of biologic-naïve patients was similar to that in the entire group of 9,135 patients with active RA. Of the biologic-naïve patients with active RA, 2,164 (53.7%) had neither elevated ESR nor CRP. Only 765 (19.0%) had elevated levels of both ESR and CRP. Among the remaining 27.3% of patients, levels of ESR and CRP were discordant: 510 (12.6%) had an elevated ESR, but not CRP, and 592 (14.7%) had an elevated CRP, but not ESR (Figure 1B).

**Figure 2:**
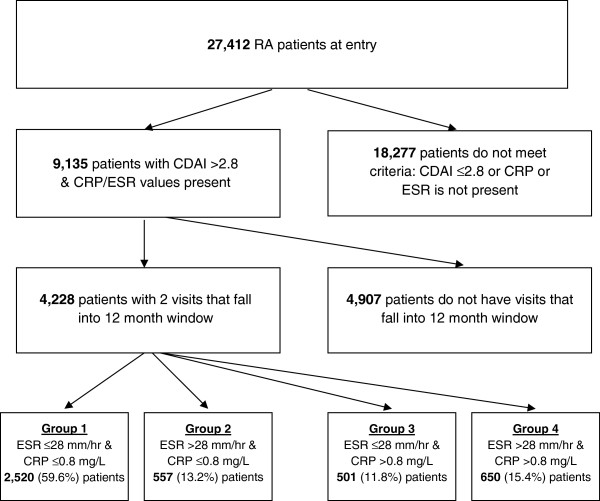
Flow chart showing the distribution of study patients.

Of the 9,135 patients who were eligible for sample inclusion, 4,228 patients had a study visit in the 60-day window of the one-year follow-up visit. The distribution of APR levels at baseline, in this subset of patients with one-year follow-up, was also similar to that in the entire group of 9,135 patients with active RA. Of the patients with active RA and a one-year follow-up visit, 2,520 (59.6%) had neither elevated ESR nor CRP. Only 650 (15.4%) had elevated levels of both ESR and CRP. Among the remaining 25.0% of patients, levels of ESR and CRP were discordant: 557 (13.2%) had an elevated ESR, but not CRP, and 501 (11.8%) had elevated CRP, but not ESR. For subsequent analyses, the 1,058 patients with discordant APR levels (ESR >28 mm/h but CRP ≤8 mg/L or CRP >8 mg/L but ESR ≤28 mm/h) were combined into a single group (Figure 2).

The baseline mHAQ and CDAI scores were lowest for patients with active RA but with neither APR elevated (Table 1). Both mHAQ and CDAI scores increased sequentially as the number of elevated APR levels increased (neither APR elevated, discordant APR levels, or both APR elevated) at baseline. Each individual component of the CDAI followed the same trend. Methotrexate (MTX) use did not differ between groups. As the number of elevated baseline APRs increased, the proportion of patients taking prednisone also increased (even after adjustment for age). These trends were also observed among the subset of biologic-naïve patients with active RA (Table 2). In contrast to prednisone use, among all 9,135 patients with active RA, the proportion of patients using biologics decreased with increasing number of elevated baseline APRs (Table 1).

**Table 1:** Baseline characteristics by APR levels

	**Neither APR elevated**	**APR levels discordant**	**Both APR elevated**	***P *** **value**
	**n = 2,520**	**n = 1,058**	**n = 650**	
Age (years)	59.29 ± 13.22	62.21 ± 13.21	62.71 ± 13.34	<0.0001
Duration of RA (years)	11.01 ± 9.83	11.93 ± 10.68	10.91 ± 10.27	0.0406
One-year follow-up duration (days)	362.63 ± 30.51	363.33 ± 31.06	363.46 ± 32.03	0.7410
mHAQ	0.33 ± 0.40	0.45 ± 0.47	0.56 ± 0.54	<0.0001
CDAI	12.89 ± 10.15	15.18 ± 11.16	20.47 ± 14.68	<0.0001
Tender joints	3.73 ± 5.24	4.29 ± 5.40	6.30 ± 7.21	<0.0001*
Swollen joints	4.04 ± 5.13	4.80 ± 5.49	6.64 ± 6.42	<0.0001*
Patient global assessment	30.91 ± 23.82	36.35 ± 25.45	42.90 ± 27.56	<0.0001
MD global assessment	20.21 ± 16.61	24.67 ± 19.06	32.42 ± 22.50	<0.0001
Prednisone use	680 (26.98%)	341 (32.23%)	275 (42.31%)	<0.0001
Methotrexate use	1,668 (66.19%)	698 (65.97%)	417 (64.15%)	0.6166
Biologic use	1,263 (50.12%)	447 (42.25%)	224 (34.46%)	<0.0001

^*^Log-linear model (Poisson) - *χ*^2^ test. APR, acute phase reactant; RA, rheumatoid arthritis; mHAQ, modified Health Assessment Questionnaire; CDAI, Clinical Disease Activity Index.

**Table 2:** Baseline characteristics of biologic-naïve patients by APR levels

	**Neither APR elevated**	**APR levels discordant**	**Both APR elevated**	***P *** **value**
	**n = 1,012**	**n = 487**	**n = 324**	
Age (years)	60.92 ± 13.49	63.24 ± 13.51	64.26 ± 12.79	<0.0001
Duration of RA (years)	8.62 ± 9.37	9.55 ± 10.46	7.47 ± 8.38	0.0074
One-year follow-up duration (days)	363.43 ± 29.79	364.78 ± 31.48	365.43 ± 31.72	0.5196
mHAQ	0.29 ± 0.38	0.38 ± 0.43	0.48 ± 0.53	<0.0001
CDAI	12.54 ± 10.18	14.61 ± 11.21	19.04 ± 14.91	<0.0001
Tender joints	3.53 ± 5.14	4.10 ± 5.46	5.68 ± 6.92	<0.0001*
Swollen joints	4.19 ± 5.41	4.76 ± 5.53	6.92 ± 6.73	<0.0001*
Patient global assessment	28.47 ± 23.70	34.31 ± 25.08	39.04 ± 27.26	<0.0001
MD global assessment	19.73 ± 15.59	23.22 ± 18.46	31.20 ± 23.03	<0.0001
Prednisone use	247 (24.41%)	138 (28.34%)	124 (38.27%)	<0.0001
Methotrexate use	696 (68.77%)	322 (66.12%)	191 (58.95%)	0.0050

^*^Log-linear model (Poisson) - *χ*^2^ test. APR, acute phase reactant; RA, rheumatoid arthritis; mHAQ, modified Health Assessment Questionnaire; CDAI, Clinical Disease Activity Index.

**Figure 3:**
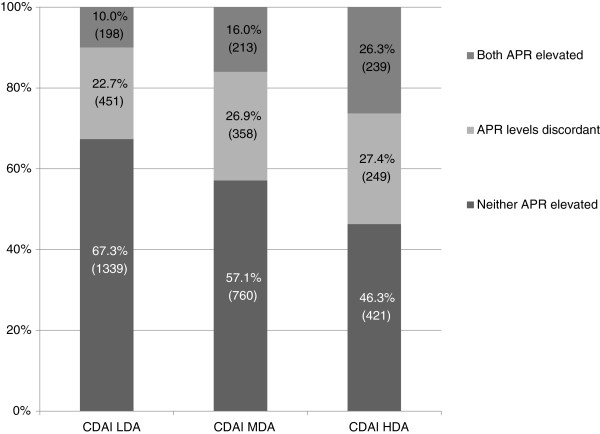
**Distribution of acute phase reactant groups among the CDAI disease activity levels at baseline.** Distribution of acute phase reactant groups among the CDAI disease activity levels at baseline in the 4,228 RA patients with a one-year follow-up visit. Percentages within the bars indicate relative proportions of subjects within each CDAI disease activity level. Numbers in parentheses within the bars enumerate subjects in each group. CDAI, Clinical Disease Activity Index; RA, rheumatoid arthritis; LDA, low disease activity; MDA, moderate disease activity; HDA, high disease activity.

Distribution of disease activity levels at baseline was significantly different (*P* <0.001) among the APR groups (Figure 3). Patients with both APRs elevated had a 36.8% rate of CDAI high disease activity, contrasted with 23.5% in those with discordant APR levels and 16.7% in those with neither APR elevated. Rates of moderate disease activity were similar among APR groups (32.8% for both APR elevated, 33.8% for discordant APR levels, and 30.2% for neither APR elevated), but rates of CDAI low disease activity differed (30.5% for both APR elevated versus 42.6% for discordant APR levels, and 53.1% for neither APR elevated). Disease activity levels at baseline were distributed similarly among the APR groups in the subset of biologic-naïve patients with active RA (data not shown).

### One-year follow-up

The mean mHAQ score at one-year follow-up was lowest for patients with active RA and neither APR elevated and was highest for those with both APRs elevated (Tables 3 and 4). Likewise, the mean CDAI score at one-year follow-up was lowest for those patients with neither APR elevated and was highest for those with both APRs elevated.

**Table 3:** Clinical and functional status at one-year follow-up by APR levels

	**Neither APR elevated**	**APR levels discordant**	**Both APR elevated**	***P *** **value**
	**n = 2,520**	**n = 1,058**	**n = 650**	
mHAQ	0.34 ± 0.42	0.42 ± 0.49	0.47 ± 0.54	<0.0001
CDAI	10.45 ± 10.74	11.34 ± 10.72	12.75 ± 11.87	<0.0001
Tender joints	3.04 ± 5.36	3.01 ± 4.88	3.55 ± 5.87	0.1125*
Swollen joints	2.93 ± 4.65	3.31 ± 4.72	4.03 ± 5.27	<0.0001*
Patient global assessment	28.50 ± 24.81	31.53 ± 26.13	31.74 ± 26.68	<0.0007
MD global assessment	16.02 ± 16.31	18.23 ± 17.19	19.73 ± 19.73	<0.0001

^*^Log-linear model (Poisson) - *χ*^2^ test. APR, acute phase reactant; mHAQ, modified Health Assessment Questionnaire; CDAI, Clinical Disease Activity Index.

**Table 4:** Clinical and functional status at one-year follow-up by APR levels among biologic-naïve patients

	**Neither APR elevated**	**APR levels discordant**	**Both APR elevated**	***P *** **value**
	**n = 1,012**	**n = 487**	**n = 324**	
mHAQ	0.28 ± 0.40	0.32 ± 0.43	0.34 ± 0.46	0.0623
CDAI	9.65 ± 10.11	9.75 ± 9.81	10.85 ± 11.17	0.2243
Tender joints	2.64 ± 4.93	2.41 ± 4.33	2.83 ± 5.17	0.4378*
Swollen joints	2.90 ± 4.66	2.91 ± 4.54	3.52 ± 5.04	0.1299*
Patient global assessment	26.23 ± 24.77	28.36 ± 25.62	28.68 ± 26.53	0.1149
MD global assessment	14.52 ± 14.82	15.61 ± 15.38	16.43 ± 15.85	<0.0001
Biologic use	150 (14.84%)	83 (17.08%)	67 (20.68%)	0.0437

^*^Log-linear model (Poisson) - *χ*^2^ test. APR, acute phase reactant; mHAQ, modified Health Assessment Questionnaire; CDAI, Clinical Disease Activity Index.

There was a positive association between the magnitude of improvement in both CDAI and mHAQ scores at one year and the number of APRs elevated at baseline. The mean mHAQ score (± SD) decreased by 0.10 ± 0.43 for those with both APRs elevated at baseline, but did not change for those patients with neither APR elevated at baseline (Table 5). The mean CDAI score (± SD) decreased by 7.68 ± 14.19 for those patients with both APRs elevated, by 3.92 ± 12.37 for those with discordant APR levels, and by only 2.40 ± 10.87 for those with neither APR elevated (Table 5). A similar trend was observed for each of the individual components of the CDAI, each of which was statistically significant at *P* <0.0001. The positive association between the magnitude of improvement in both CDAI and mHAQ scores at one year and the number of APRs elevated at baseline was also observed in the subset of biologic-naïve RA patients, as was the similar trend for each of the individual components of the CDAI (Table 6).

**Table 5:** Change in clinical and functional status between baseline and one year by APR levels

	**Neither APR elevated**	**APR levels discordant**	**Both APR elevated**	***P *** **value**
	**n = 2,520**	**n = 1,058**	**n = 650**	
mHAQ	0.01 ± 0.31	-0.03 ± 0.40	-0.10 ± 0.43	<0.0001
CDAI	-2.40 ± 10.87	-3.92 ± 12.37	-7.68 ± 14.19	<0.0001
Tender joints	-0.69 ± 5.48	-1.28 ± 5.81	-2.75 ± 6.98	<0.0001
Swollen joints	-1.12 ± 5.11	-1.49 ± 5.53	-2.58 ± 5.95	<0.0001
Patient global assessment	-2.38 ± 25.21	-4.90 ± 27.78	-11.29 ± 30.11	<0.0001
MD global assessment	-4.21 ± 17.30	-6.45 ± 19.78	-12.57 ± 23.19	<0.0001

APR, acute phase reactant; mHAQ, modified Health Assessment Questionnaire; CDAI, Clinical Disease Activity Index.

**Table 6:** Change in clinical and functional status between baseline and one year by APR levels among biologic-naïve patients

	**Neither APR elevated**	**APR levels discordant**	**Both APR elevated**	***P *** **value**
	**n = 1,012**	**n = 487**	**n = 324**	
mHAQ	0.00 ± 0.29	-0.06 ± 0.35	-0.14 ± 0.41	<0.0001
CDAI	-2.91 ± 10.92	-4.93 ± 12.35	-8.16 ± 13.53	<0.0001
Tender joints	-0.91 ± 5.54	-1.69 ± 5.88	-2.82 ± 6.64	<0.0001
Swollen joints	-1.28 ± 5.11	-1.85 ± 5.60	-2.81 ± 5.78	<0.0001
Patient global assessment	-2.14 ± 26.41	-6.00 ± 27.96	-10.58 ± 30.18	<0.0001
MD global assessment	-5.23 ± 16.46	-7.62 ± 19.49	-14.62 ± 22.91	<0.0001

APR, acute phase reactant; mHAQ, modified Health Assessment Questionnaire; CDAI, Clinical Disease Activity Index.

## Discussion

In this study, we examined the relationship between APRs and clinical outcome measures in patients with active RA. The traditional expectation is that RA patients with CDAI high disease activity might be more likely to have levels of both APRs elevated, whereas those with CDAI low disease activity might not have elevation of either APR. However, both APRs were elevated in only 26.3% of patients with CDAI high disease activity in our cohort, whereas neither APR was elevated in 46.3%; conversely, 32.7% of those with CDAI low disease activity had at least one APR elevated and 10.0% had both APRs elevated (Figure 3). These findings imply that APR levels, which traditionally are measured in most randomized clinical trials, often do not reflect disease activity as measured by joint counts and global assessments. When only one APR was assessed at the initial evaluation, we observed that an elevated level might not be detected in up to 73.7% (670) of the patients with high CDAIs and up to 84.0% (1,118) of those with moderate CDAIs. We have thus determined that the yield of identifying an elevated APR in patients with active RA is greater when both the ESR and CRP are obtained at the baseline evaluation.

Discordance between APRs has also been observed in other studies that correlated clinical characteristics with laboratory findings [17,19,20]. Graf and colleagues found a weak correlation between CRP levels and CDAI scores (r = 0.18, *P* = 0.027) among 151 patients with RA [19]. Although all seven of their patients who were in CDAI remission had CRP <8 mg/L, only 46 (33.3%) of the 138 patients with CDAI >2.8 had CRP >8 mg/L. Crowson and coworkers also found a weak correlation between APRs and CDAI scores (r = 0.29 for CRP and r = 0.28 for ESR) among 1,247 RA patients [17]. DAS28, which includes an APR in its scoring, correlated more closely with APRs, (r = 0.48 for CRP and r = 0.53 for ESR), as expected. In another study of 689 patients with RA, Wolfe and collaborators found 41% agreement between DAS28 and ESR (Kendall's tau-a 0.41; 95% CI: 0.37 to 0.45) [20].

In our cohort of patients with active RA, as defined by CDAI >2.8, the number of elevated APRs (0, 1, or 2) often did not reflect disease activity at the time of the baseline measure: 46.3% had neither APR elevated with a high CDAI and 32.7% had elevation of at least one APR, despite being in a CDAI low disease activity state (Figure 3). Both APRs were elevated in only 26.3% of those with high baseline CDAIs. Crowson and coworkers also observed a poor correlation between the number of swollen joints and APR elevation [17]. Discordance between clinical parameters and laboratory values may be due, at least in part, to the lower sensitivity of detecting subtle joint inflammation during the clinical examination of patients with RA [27]. APR elevation may also reflect inflammation in areas other than the joints [28]. Thus, in many RA patients, APR values alone may not be accurate indicators of disease activity.

We observed an association between the numbers of APR elevated at baseline and the CDAI or mHAQ response at one year. The greatest decrease in CDAI was observed when both APRs were elevated at baseline and the smallest when neither APR was elevated. An intermediate decrease was observed when APR levels were discordant. Similar ‘APR-dependent’ associations were found between each of the individual components of CDAI, except for the tender joint count (Tables 5 and 6). The number of tender joints can be influenced by factors other than joint inflammation, including osteoarthritis of the examined joints or pain amplification in central sensitization syndromes, thereby rendering it less reflective of RA disease activity [29]. The change in mHAQ score also correlated with the number of APRs elevated at baseline, although less consistently than did the change in CDAI score (Tables 5 and 6).

As expected, there was an inverse relationship between APRs and the use of biologics. However, there was a direct relationship between APRs and the use of prednisone: the greatest proportion of patients using prednisone was in the group of patients with both APRs elevated. Thus, the relationships of APRs to the use of prednisone and to the use of biologics move in opposite directions. This may reflect both the effectiveness of biologics in reducing APR levels and the propensity of physicians to use corticosteroids to treat patients with active RA. No difference was observed among patients taking MTX, consistent with the previously reported weak effect of MTX on APRs [30,31].

This was an observational study of data from a registry, in which clinical measurements were mandated but laboratory studies were not and were ordered according to physician practice style. Thus, not all patients in the CORRONA database had values available for both ESR and CRP. However, the demographic characteristics of the patients who did not have either APR measured were similar to those for whom an ESR or CRP level had been obtained. Among 92,062 visits for 17,445 RA patients in the CORRONA database, an APR was measured at 47,164 (51.2%) of the visits. There was no association between whether or not an APR level had been assessed and age, sex, or measures of disease activity or physical function [32].

For our study, we chose cutoff values of 28 mm/h for ESR and 8 mg/L for CRP in patients with active RA, since these APR values have typically been used as entry criteria for clinical trials of novel therapies for RA. However, the cutoff value for the ESR is above the upper limit of normal used by clinical laboratories for this test. Moreover, in clinical practice, rheumatologists prefer to see APR levels well below the upper limit of normal when treating patients with RA. Thus, the proportion of patients with 'normal’ levels of both APR might have been smaller if a lower cutoff value for the ESR had been used. However, our finding that disease activity measures correlate weakly with APR elevation underscores the limitations of using minimum values of 28 mm/h for ESR or 8 mg/L for CRP, in addition to swollen and tender joint counts, as entry criteria for clinical trials of novel therapies for RA, since the majority of patients with active RA have levels of both ESR and CRP below these threshold values. In addition, there was a possible floor effect among patients with fewer APRs elevated at baseline: patients who are doing better have less 'room for improvement’ and those who start from a 'worse place’ may make greater gains.

For most patients in our study, CDAI was responsive to change and can serve as a good indicator of the degree of joint inflammation. A better understanding of the relationship between APRs and disease activity, as measured by the CDAI, will allow for a more informed use of these measures in clinical practice and in clinical trials of novel therapeutic agents. In addition, our observation that elevation of APR levels is often discordant with what clinicians can measure in the clinic implies that systemic inflammation may continue, even while joint swelling and tenderness are 'controlled’. It is also possible that this systemic inflammation involves areas other than the joints [28].

Normalization of CRP levels in subjects at risk for heart disease is associated with a significantly lower number of cardiovascular events [28]. This same comorbidity is the chief cause of premature mortality in patients with RA [33]. It therefore follows that elucidating the long-term relationship between APRs and cardiovascular disease in patients with RA will add greatly to our understanding of the overall relevance of these measures in clinical domains other than those typically measured in daily practice and in randomized clinical trials in rheumatology. These relationships likely will become clearer with continued observation of these variables among large numbers of patients followed in long-term disease registries.

## Conclusions

Obtaining both ESR and CRP in a patient with active RA increases the yield of identifying an elevated APR level. However, APR levels often do not reflect disease activity, as measured by joint counts and global assessments. Change in CDAI and mHAQ at one year is associated with the number of APRs elevated at baseline.

The normal APR levels present in over half of patients with active joint disease, as determined by CDAI, in a large United States registry of RA patients would exclude these patients from participation in many clinical trials of novel therapies for RA. Thus, clinical trials of novel therapies for RA might use CDAI >10 (the cutoff for moderate disease activity), rather than elevated APR, as a criterion for entry. These changes might have the effect of increasing enrollment while providing a more representative population of patients for clinical trials. The long-term implications of discordance in APRs will need to be studied further in large populations of RA patients followed prospectively over prolonged time intervals.

## Abbreviations

APR: acute phase reactant; CDAI: Clinical Disease Activity Index; CORRONA: Consortium of Rheumatology Researchers of North America; CRP: C-reactive protein; DAS: Disease Activity Score; DMARD: disease-modifying antirheumatic drug; ESR: erythrocyte sedimentation rate; HAQ-DI: Health Assessment Questionnaire Disability Index; mHAQ: modified Health Assessment Questionnaire; MTX: methotrexate; RA: rheumatoid arthritis; SDAI: Simplified Disease Activity Index.

## Competing interests

The authors declare that they have no competing interests.

## Authors’ contributions

JK conceived of the study, participated in its design and coordination and in analysis and interpretation of data, and drafted the manuscript. OM participated in the coordination of the study and helped to draft the manuscript. SPM participated in the design of the study, performed the statistical analysis, and helped to draft the manuscript. JMK and JDG organized and participated in data acquisition and coordination of the study and helped to revise the manuscript critically for important intellectual content. GWR and EMG participated in the analysis and interpretation of data and helped to revise the manuscript critically for important intellectual content. DEF participated in the conception, design and coordination of the study and in analysis and interpretation of data, and helped to draft the manuscript. All authors read and approved the final manuscript.
